# Supplementary figures and images for: A PCR plus restriction enzyme-based technique for detecting target-enzyme mutations at position Pro-106 in glyphosate-resistant Lolium perenne
Source: PLoS One. 2021 Feb 2;16(2):e0246028. doi: 10.1371/journal.pone.0246028 (PMC7853469; doi:10.1371/journal.pone.0246028)

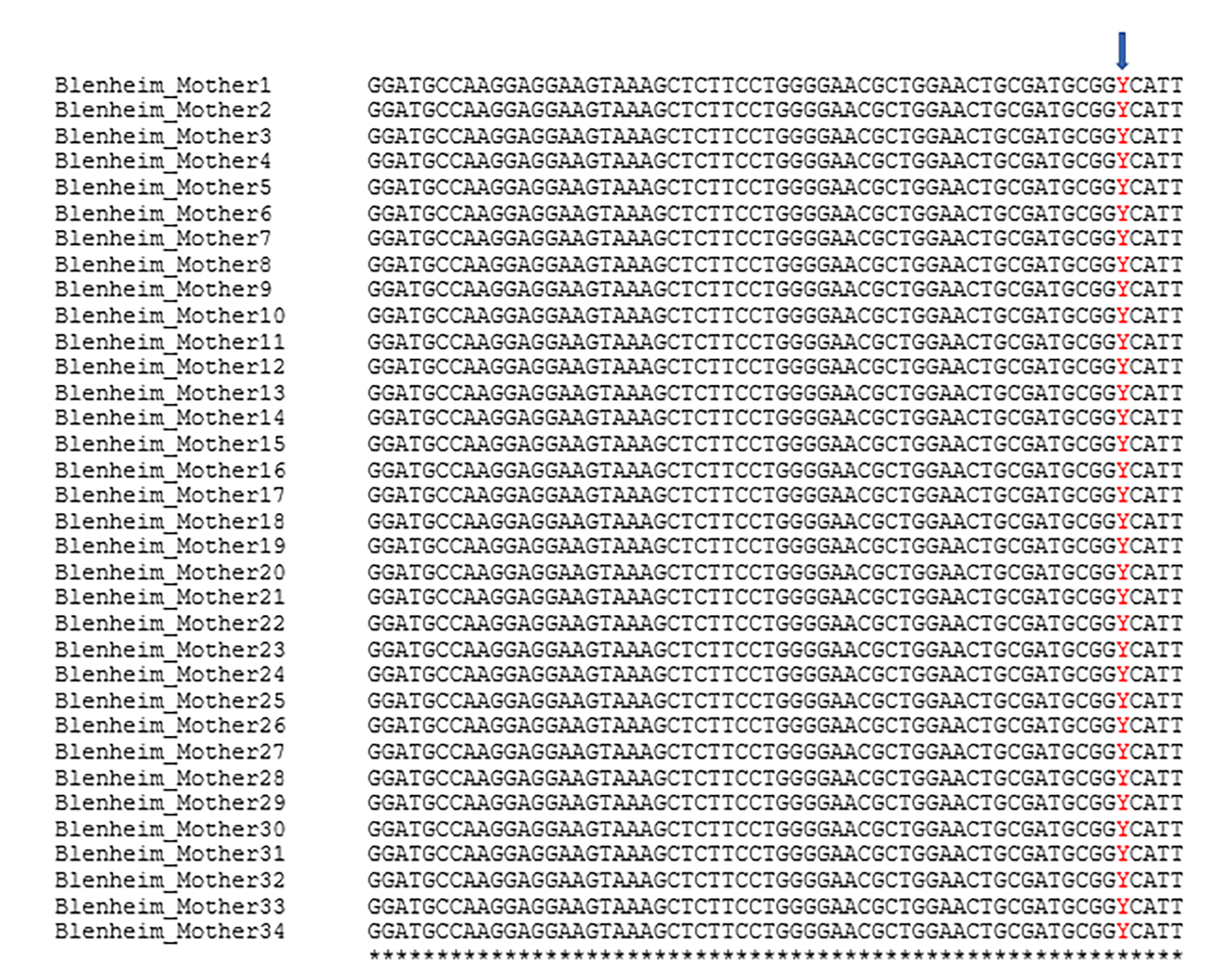

Supplement: S1 Fig — The arrow shows the SNP mutation conferring resistance at codon 106 in the EPSPS gene. As both alleles in the individual were sequenced simultaneously a heterozygous state at the first base of codon 106 (arrow) identified as a pyrimidine (Y) comprising the wild type glyphosate sensitive C nucleotide and the glyphosate-resistant T mutation at this position. (TIF) [file pone.0246028.s001.tif]
