# Supplementary material for: A PCR plus restriction enzyme-based technique for detecting target-enzyme mutations at position Pro-106 in glyphosate-resistant Lolium perenne
Source: PLoS One. 2021 Feb 2;16(2):e0246028. doi: 10.1371/journal.pone.0246028 (PMC7853469; doi:10.1371/journal.pone.0246028)
Supplement: S1 Raw images — (PDF) [file pone.0246028.s002.pdf]

Fig. 1

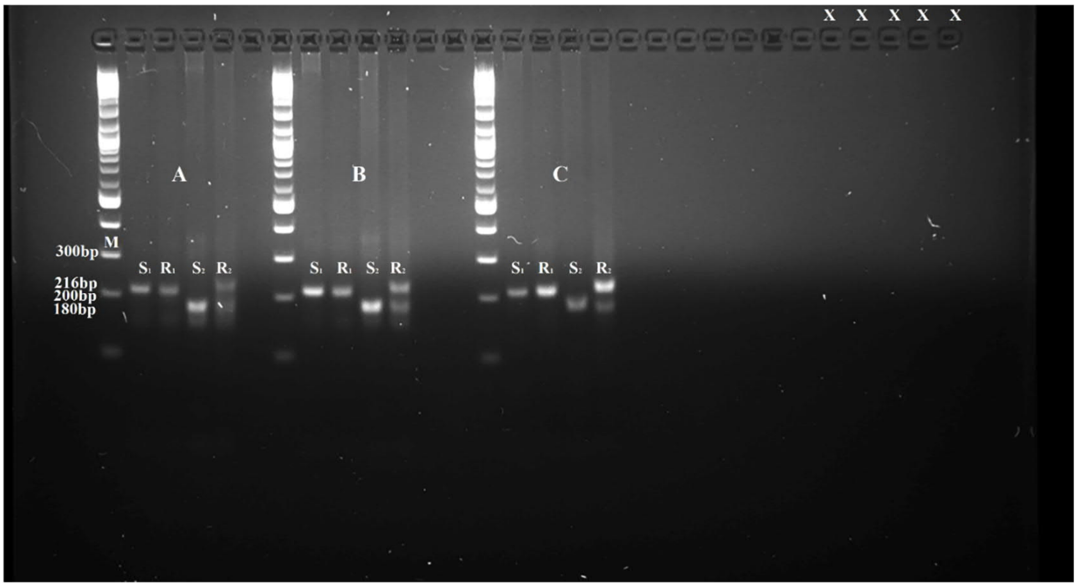

The digestion patterns for three restriction enzymes, *Msc*I (A), *Sau*96I (B) and *Nla*IV (C) in individuals of glyphosate-susceptible (S) and glyphosate-resistant (R) populations. S_1 and R_1 represent undigested samples showing the 216 bp (base pair) amplicon R and S populations, respectively. S_2 represents restriction enzyme-digested samples (180 bp) for glyphosate-susceptible population. R_2 represents restriction enzyme-digested samples (216 + 180 bp) for a heterozygous glyphosate-resistant population. **M** denotes the DNA size standard lanes showing the 200 and 300 bp fragments of the 1 kb Plus size marker ladder (NEB, UK). X donates any lanes not included in the final figures. The image was prepared by loading products on an agarose 1x LB (lithium borate) 2% (w v^{-1}) gel containing 0.5 µg ml^{-1} ethidium bromide before they were run at 4 V cm^{-1} for 1.5 h and visualised under UV illumination using a Gel Doc XR 2000 system (Bio-Rad Laboratories).

Fig. 3

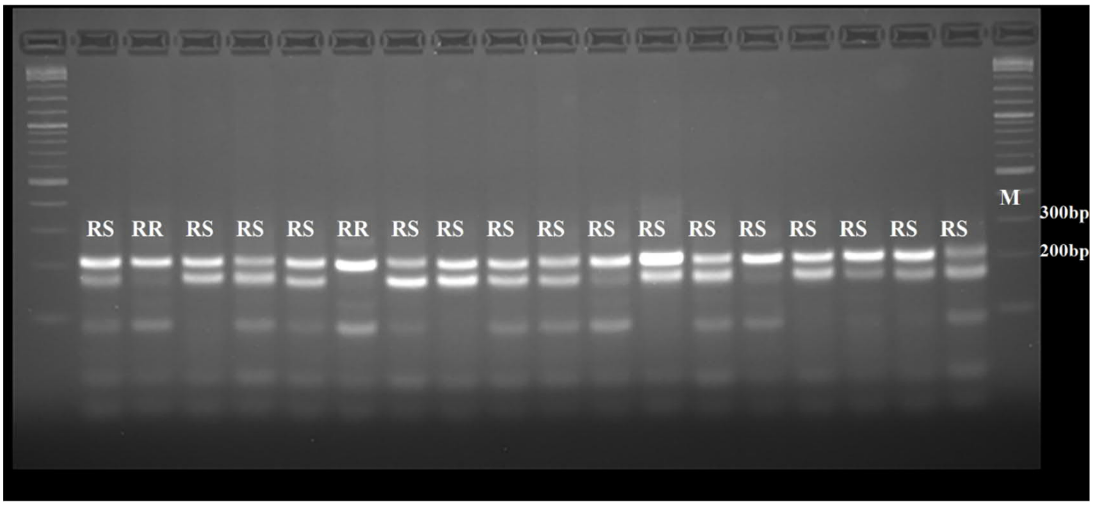

The digestion patterns for the restriction enzyme, *Sau*96I for detecting mutations at codon 106 in 18 individuals from a glyphosate-resistant Blenheim population. RS and RR represent digested samples for glyphosate-resistant individuals that were heterozygous and homozygous, respectively, for single nucleotide polymorphism mutations conferring resistance at codon 106 in the *EPSPS* gene. **M** denotes the DNA size standard lanes showing the 200 and 300 bp fragments of the 1 kb Plus size marker ladder (NEB, UK). The image was prepared by loading products on an agarose 1x LB (lithium borate) 2% (w v^{-1}) gel containing 0.5 µg ml^{-1} ethidium bromide before they were run at 4 V cm^{-1} for 1.5 h and visualised under UV illumination using a Gel Doc XR 2000 system (Bio-Rad Laboratories).
