# Supplementary material for: A PCR plus restriction enzyme-based technique for detecting target-enzyme mutations at position Pro-106 in glyphosate-resistant Lolium perenne
Source: PLoS One. 2021 Feb 2;16(2):e0246028. doi: 10.1371/journal.pone.0246028 (PMC7853469; doi:10.1371/journal.pone.0246028)
Supplement: Attachment — Submitted filename: Response to Reviewers.docx [file pone.0246028.s003.docx]

All the modifications have been indicated using the track changes feature in MS Word. Detailed responses to the reviewers are:

**Editorial requirements**

1- Please ensure that your manuscript meets PLOS ONE's style requirements, including those for file naming.

**Response:** We have formatted our manuscript according to PLOS ONE`s style requirements.

2- We note that you have included the phrase “data not shown” in your manuscript. Unfortunately, this does not meet our data sharing requirements. PLOS does not permit references to inaccessible data. We require that authors provide all relevant data within the paper, Supporting Information files, or in an acceptable, public repository. Please add a citation to support this phrase or upload the data that corresponds with these findings to a stable repository (such as Figshare or Dryad) and provide and URLs, DOIs, or accession numbers that may be used to access these data. Or, if the data are not a core part of the research being presented in your study, we ask that you remove the phrase that refers to these data.

**Response:** The authors agreed to submit the partial sequence of EPSPS gene as a supplementary file (S1 Fig) as we believe it would be more convenient for the readers to access the data when they are available as supplementary materials.

3. PLOS ONE now requires that authors provide the original uncropped and unadjusted images underlying all blot or gel results reported in a submission’s figures or Supporting Information files. This policy and the journal’s other requirements for blot/gel reporting and figure preparation are described in detail at https://journals.plos.org/plosone/s/figures#loc-blot-and-gel-reporting-requirements and https://journals.plos.org/plosone/s/figures#loc-preparing-figures-from-image-files. When you submit your revised manuscript, please ensure that your figures adhere fully to these guidelines and provide the original underlying images for all blot or gel data reported in your submission. See the following link for instructions on providing the original image data: <https://journals.plos.org/plosone/s/figures#loc-original-images-for-blots-and-gels>.

**Response:** The authors agreed to submit the original uncropped figures. Thus, we provided the original images of gels for the revised version of manuscript.

4. Please include a caption for figure 4.

**Response:** A caption has been included for Fig 4.

5. Please ensure that you refer to Figure 4 in your text as, if accepted, production will need this reference to link the reader to the figure.

**Response:** A sentence was added (Lines 272-275) referring to Fig 4.

**Reviewer**

Firstly, we would like to thank this reviewer for their comments. This reviewer requested a few modifications and they were addressed as below:

In table 1, the MscI cut site should be TGG/CCA.

**Response:** Thanks for spotting this typo. This has been modified.

Legends in figures 1 and 3, lines 446 and 467, the term “company” should be specified.

**Response:** The term has been specified. The name of the company where the ladder was purchased was added.

In figure 3, “RR” glyphosate-resistant individuals (lanes 11 and 14) seem to have the cleaved (~180 bp) dCAPS amplicon, although the bands are faint. The authors may want to elaborate it a bit in the text.

**Response:** Thanks for spotting this typo in the figure. This has been modified.

There are 4 figures in the manuscript. Figure 4 (chromatogram) did not have legend and was not mentioned in the text.

**Response:** A caption has been included for Fig 4 and a sentence was added (Lines 272-275) referring to Fig 4.
